# Comparing inhaled colistin with inhaled fosfomycin/tobramycin as an adjunctive treatment for ventilator‐associated pneumonia: An open‐label randomized controlled trial

**DOI:** 10.1111/crj.13594

**Published:** 2023-02-12

**Authors:** Atousa Hakamifard, Abbas Ali Torfeh Esfahani, Alireza Homayouni, Farzin Khorvash, Behrooz Ataei, Saeed Abbasi

**Affiliations:** ^1^ Department of Infectious Diseases, School of Medicine Isfahan University of Medical Sciences Isfahan Iran; ^2^ Infectious Diseases and Tropical Medicine Research Center Shahid Beheshti University of Medical Sciences Tehran Iran; ^3^ Research and Development Department Goldaru Pharmaceutical Company Isfahan Iran; ^4^ Department of Pharmaceutics, School of Pharmacy Isfahan University of Medical Sciences Isfahan Iran; ^5^ Nosocomial Infection Research Center Isfahan University of Medical Sciences Isfahan Iran; ^6^ Anesthesiology and Critical Care Research Center Isfahan University of Medical Sciences Isfahan Iran

**Keywords:** *Acinetobacter*, aminoglycosides, colistin, fosfomycin, nosocomial pneumonia, tobramycin

## Abstract

**Purpose:**

Although investigations are limited, adjunctive aerosolized antibiotics have been advised in the setting of gram‐negative ventilator‐associated pneumonia (VAP). This study aimed to compare the efficiency of inhaled colistin with inhaled fosfomycin/tobramycin in treating VAP due to extensively drug‐resistant (XDR) *Acinetobacter baumannii* .

**Methods:**

This single center open‐label randomized controlled trial included 60 patients who developed XDR *A. bumannii* VAP. Eligible participants were randomly assigned to two groups (no. 30). Regardless of the assignment, all participants received meropenem (2 g as a 3‐h extended infusion every 8 h) plus intravenous colistin (a loading dose of 9 million IU and then 4.5 million IU every 12 h). The control group was given inhaled colistin (1 million IU every 8 h), and the case group received inhaled tobramycin/fosfomycin (300 mg every 12 h/80 mg every 12 h) as adjunctive therapy. The primary outcome was treatment duration, and the secondary outcomes were Clinical Pulmonary Infection Score (CPIS) trend and mortality rate in the groups. The decision to stop treatment was made by the treating physician.

**Results:**

The mean treatment duration was 13.73 ± 3.22 days in the colistin group and 10.85 ± 2.84 days in the tobramycin/fosfomycin group; the mean treatment duration in the latter group was lower significantly (*P* = 0.001). CPIS was decreased in the groups significantly (*P* < 0.001), but the mean changes of CPIS were significantly different between the groups, and in the inhaled tobramycin/fosfomycin group, a greater reduction (*P* = 0.005) was observed. Two (6.67%) patients in the control group and three (10%) patients in the case group died.

**Conclusion:**

The use of inhaled tobramycin/fosfomycin in cases with XDR *A. bumannii* VAP was associated with a shorter treatment duration in this open‐label trial.

## Graphical abstract

Studies on adjunctive inhaled antibiotics for the treatment of XDR A. bumannii VAP are limited. This open‐lable randomized controlled trial focused on the efficacy of inhaled colistin and inhaled fosfomycin/tobramycin for the treatment of VAP caused by XDR A. bumannii. The use of inhaled tobramycin/fosfomycin leads to increased recovery rate and reduced duration of treatment in patients with XDR A. bumannii VAP.

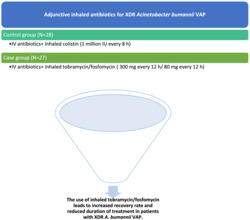

## 1 INTRODUCTION

Multidrug‐resistant (MDR) or extensively drug‐resistant (XDR) *Acinetobacter baumannii* is among the toughest antimicrobial‐resistant gram‐negative bacilli to be treated. This organism is often involved in nosocomial infections and leads to various infections, such as pneumonia and sepsis. Numerous studies have reported that *A. baumannii* is mostly MDR and, in some cases, resistant to all antibiotics.^1^ In intensive care units (ICUs), the prevalence of pneumonia caused by *Acinetobacter* species has been mentioned in several studies.^2^, ^3^

Ventilator‐associated pneumonia (VAP), a type of hospital‐related pneumonia, is among the most commonly detected infection in ICUs. It is characterized as a pulmonary parenchyma infection in cases exposed to invasive mechanical ventilation for at least 48 h, with a high mortality and morbidity rate. VAP is reported to affect 5–40% of patients, with variations depending upon several factors.^4^, ^5^

Timely treatment with appropriate antibiotics is crucial and improves survival in patients with VAP.^5^ Although treatment of VAP is usually with intravenous antibiotics, some studies have shown the benefits of inhaled antibiotics, especially in the drug‐resistant organisms. These antibiotics directly target airway and lung parenchyma resulting in high local concentrations and higher clinical responses and can be used to decrease intravenous antibiotic doses to minimize toxicities.^6^ The Infectious Diseases Society of America (IDSA) guidelines for nosocomial pneumonia recommend the addition of inhaled antibiotics to intravenous antibiotics for patients with gram‐negative pneumonia due to resistant organisms, sensitive only to polymyxins and aminoglycosides.^7^ In several studies, colistin and aminoglycosides such as tobramycin are considered among the most common inhaled antibiotics.^8^, ^9^, ^10^, ^11^, ^12^

Colistin is one of the choices for treating resistant *A. baumannii* strains. This agent is a nephrotoxic antibiotic, and the use of inhaled form of colistin reduces the incidence of this adverse effect.^13^

Tobramycin is an aminoglycoside antibiotic that inhibits protein synthesis, and inhaled form has been used for VAP.^12^ Fosfomycin is a wide‐ranging spectrum antibiotic with in vitro activity against both gram‐positive and gram‐negative bacterial organisms. This agent prevents the synthesis of the bacterial cell wall, increases the absorption of tobramycin, and leads to increased inhibition of protein synthesis and ultimately the killing of bacteria.^14^, ^15^ Several in vitro investigations have proposed a potential role for intravenous fosfomycin in treating MDR *A. bumannii*. Given the synergistic impact of fosfomycin and colistin in the in vitro investigations, it might be an efficient adjunctive treatment.^16^, ^17^ Also, inhaled fosfomycin/tobramycin in cases with cystic fibrosis with *Pseudomonas* airway infection has been studied.^18^

Although various studies have reported the effect of inhaled use of the antibiotics colistin and tobramycin in the VAP treatment, the effect of using the inhaled fosfomycin/tobramycin combination in treating XDR *A. baumannii*‐caused VAP has not been studied. According to unavailability of intravenous fosfomycin in our country and considering the synergistic effect of fosfomycin/tobramycin combination, it seems that the use of combination of inhaled tobramycin/fosfomycin along with intravenous antibiotics can be useful. This study was performed to compare the impact of inhaled colistin and fosfomycin/tobramycin in treating VAP due to XDR *A. bumannii* being sensitive only to colistin.

## 2 MATERIAL AND METHODS

### 2.1 Trial design and participants

A single‐center open‐label randomized controlled trial was performed from 2019 to 2020 at Al‐Zahra hospital, a referral teaching hospital affiliated to Isfahan University of Medical Sciences, Iran. The ethics committee of Isfahan University of Medical Sciences approved the trial with No: IR.MUI.MED.REC.1398.405. It was carried out in accordance with the Helsinki Declaration and was recorded in the Iranian clinical trials registry (www.irct.ir; N: IRCT20171230038142N11). Prior to any research processes, all legal representatives provided written informed consent. A designed questionnaire was used to obtain patient data from medical files, hospital databases, or clinical documents. The inclusion criteria were females and males with range age between 20 and 85 years, signs and symptoms compatible with VAP diagnosis, and endotracheal aspiration (ETA) culture positive for XDR *A. bumannii*. Death of the patient before obtaining the culture result, changing the treatment protocol, need for additional antibiotic therapy, and dissatisfaction with continuing experimental treatment were considered as exclusion criteria.

### 2.2 Definitions

#### 2.2.1 VAP

VAP is characterized as pneumonia developing more than 48 h after intubation and mechanical ventilation. Diagnosis of this entity necessitates a high level of clinical suspicion, radiographic examination, and microbiologic investigation of respiratory secretions.^19^ Clinical criteria for the suspicious of VAP include a new or progressive radiographic infiltrate and at least two of three following clinical characteristics: fever above 38°C, leukopenia or leukocytosis, and purulent secretions. These represent the most accurate criteria for starting empiric antibiotic therapy.^20^ The trend of Clinical Pulmonary Infection Score (CPIS) was evaluated,^21^ and chest X‐ray, ETA secretions and cultures, temperature, and leukocyte and PaO_2_/FiO_2_ percentage were also recorded at specified time intervals: at the initiation of treatment, after 72 h and 7 days, and at the treatment termination.

#### 2.2.2 MDR, XDR, and pandrug resistance

Definitions for drug‐resistant organisms are as follows: MDR refers to nonsusceptibility to equal or more than one agent in equal and more than three effective antimicrobial categories. XDR refers to nonsusceptibility to equal or more than one agent in all but equal or less than two antimicrobial categories. Nonsusceptibility to all antimicrobial agents tested was defined as pandrug resistance.^22^ The inclusion criteria in this study were XDR *A. bumannii*.

### 2.3 Fosfomycin preparation

In order to obtain fosfomycin solution, the fosfomycin disodium salt was accurately weight and dissolve in distilled water under laminar air flow condition to prepared the 4% (w/v) concentration. The disodium salt of fosfomycin is more appropriate than other salt regarding to it more solubility in order to nebulization. In order to adjust the pH of solution to 7, small amount of HCl were added to the solution. Moreover, the solution was filtered throw 0.1 sterile micrometer to obtained sterile solution. This study employed 2 mL of this solution, which is equal to 80 mg of the medicine, to benefit from the right dose of the medicine. This amount of drug entered the nebulizer jet chamber as a solution and was used by patients. The adjustment of tonicity is not needed as the solution diluted with vapor water during nebulization and does not cause irritation when inhaled. Tobramycin nebulization was performed using 300 mg/5 mL solutions available on the market. This solution was applied after nebulization of fosfomycin.

### 2.4 Treatment protocol

Following VAP diagnosis, empiric antibiotic courses were commenced based on clinical judgment by infectious diseases specialist and were later changed based on ETA culture results. A total of 60 eligible patients were recruited for the study and were randomly assigned to two groups of 30 using random allocation software. Regardless of group assignment, all participants received meropenem (2 g as a 3‐h extended infusion every 8 h) and intravenous colistin (a loading dose of 9 million IU and then 4.5 million IU every 12 h). The control group was given inhaled colistin (1 million IU per 8 h), whereas the case group was given inhaled tobramycin/fosfomycin (300 mg every 12 h/80 mg every 12 h). The criteria for discontinuation of treatment included cessation of fever, resolution of leukocytosis, reduction of lung secretions, or improvement of Chest X‐ray (CXR) changes. Reduction of CPIS also was used. The decision to stop treatment was made by the treating physician.

### 2.5 Outcome assessment

Duration of treatment was the primary outcome, and the CPIS trend at determined time intervals and mortality rate were as the secondary outcomes among the groups.

### 2.6 Statistical analysis

The data were analyzed using SPSS software version 26 by Chi‐square (to compare qualitative data between the two groups), *t*‐test, *t*‐paired (for comparison of quantitative variables between the two groups), and repeated measures analysis of variance (to compare mean changes in variables among the groups). *P* < 0.05 was regarded as a significant level.

## 3 RESULTS

**FIGURE 1:**
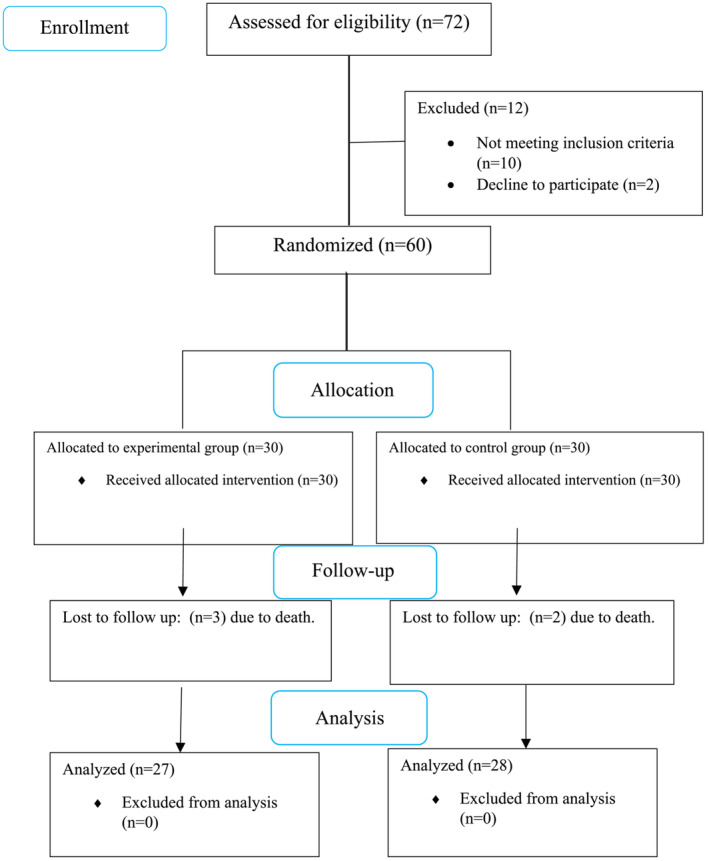
The study's CONSORT flowchart.

In this research, 60 individuals with XDR *A. baumannii* VAP were divided into two groups of 30 and given inhaled colistin or tobramycin/fosfomycin. During the intervention, two cases in the inhaled colistin group and three cases in the inhaled fosfomycin/tobramycin group were excluded from the study due to death before the end of the treatment period (Figure 1).

In terms of age and gender distribution, the reason for ICU admission, underlying diseases, latest antibiotic consumption, and past ICU admission history, the two groups did not significantly differ (Table 1). Also, the interval between hospitalizations in the ICU and the diagnosis of VAP, as well as the ventilation duration until VAP, did not significantly differ among the groups. None of the patients had a previous history of colistin use.

**TABLE 1:** Distribution of demographic characteristics and clinical history of subjects in the groups.

Variables		Groups		*P*‐value
		Inhaled colistin (*n* = 28)	Inhaled tobramycin/fosfomycin (*n* = 27)	
Mean (±*SD*) of age (year)		63.6 ± 14.9	59.3 ± 15.2	0.29
Gender *n* (%)	Male	15 (53.6)	18 (66.7)	0.32
	Female	13 (46.4)	9 (33.3)	
Cause of hospitalization in the intensive care unit	cerebral vascular accident (CVA)	13 (46.4)	15 (55.6)	0.19
	Multiple trauma	2 (7.1)	4 (14.8)	
	Respiratory distress (excluding pneumonia)	5 (17.9)	6 (22.2)	
	Iintracerebral hemorrhage (CH)	3 (10.7)	2 (7.4)	
	Other	5 (17.9)	0 (0)	
Presence of underlying diseases		26 (92.9)	23 (85.2)	0.36
Recent use of antibiotics		23 (82.1)	25 (92.6)	0.25
Previous using of carbapenem		13 (46.4)	15 (55.6)	0.498
History of previous ICU hospitalization		10 (35.7)	11 (40.7)	0.7
Mean of days between ICU admission to VAP diagnosis		17.6 ± 22.4	15.3 ± 9.7	0.63
Duration of mechanical ventilation before VAP incidence		19.5 ± 4.6	15.7 ± 9.8	0.45

Abbreviations: ICU, intensive care unit; VAP, ventilator‐associated pneumonia.

**FIGURE 2:**
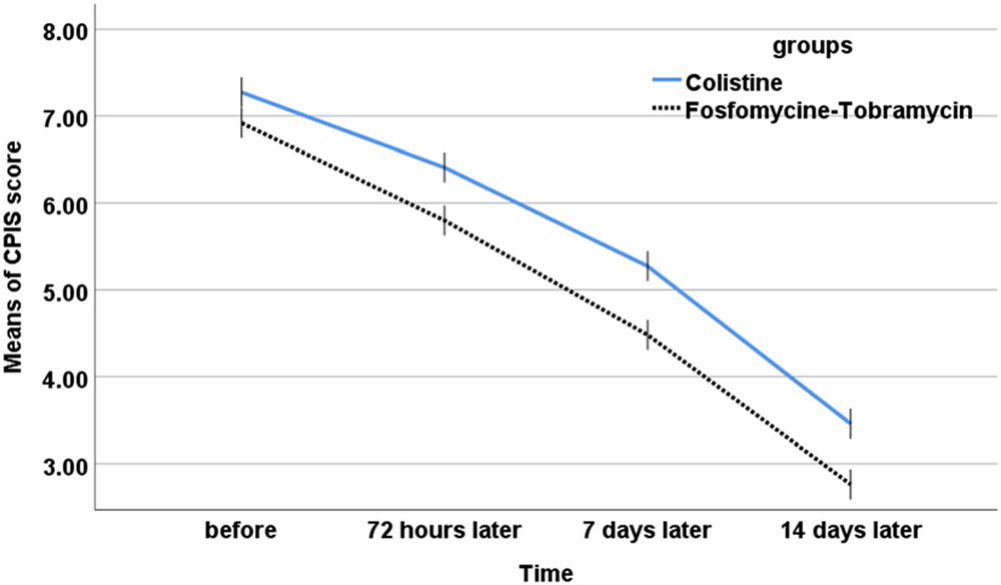
CPIS changes trend during treatment among the groups. CPIS, Clinical Pulmonary Infection Score.

The mean CPIS did not differ between the groups at the start of treatment; however, at 72 h treated with tobramycin/fosfomycin, 7 days later, and at the treatment termination, the mean score in the tobramycin/fosfomycin group was significantly lower. Also, in intragroup examinations, the CPIS in both groups was significantly reduced (*P* < 0.001). The mean CPIS changes differed significantly among the two groups, with the inhaled tobramycin/fosfomycin group experiencing a higher drop (*P* = 0.005) (Figure 2 and Table 2).

**TABLE 2:** Mean and standard deviation of Clinical Pulmonary Infection Score over the treatment period of the groups.

Time	Groups		*P*‐value
	Inhaled colistin (*n* = 28)	Inhaled tobramycin/fosfomycin (*n* = 27)	
At the time of diagnosis	7.21 ± 0.69	7.04 ± 0.85	0.399
72 h later	5.46 ± 0.74	5.93 ± 0.96	0.024
7 days later	5.38 ± 1.36	4.54 ± 1.07	0.016
At the end of the treatment	3.45 ± 0.8	2.76 ± 0.83	0.006
*P*‐value**	<0.001	<0.001	0.005***

* Significant level of difference among the groups in each time period according to the *t*‐test.

** Significant level of difference within each group on the basis of repeated measures ANOVA.

*** Significant level of difference between the two groups based on repeated measures ANOVA.

According to the results, the mean levels of C‐reactive protein (CRP) and procalcitonin in the treatment dimension in both groups were significantly reduced compared with the beginning of treatment; however, their changes trend did not significantly differ between the groups. Mean creatinine and urea levels at the beginning and end of the study did not significantly differ among the groups (Table 3).

**TABLE 3:** Mean and standard deviation of serum procalcitonin, urea, and creatinine levels in the two groups.

Variables	Time	Groups		*P*‐value*
		Inhaled colistin (*n* = 28)	Inhaled tobramycin/fosfomycin (*n* = 27)	
CRP	At the start of treatment	69 ± 23	72.9 ± 28	0.58
	At the end of treatment	48.1 ± 21.4	49.3 ± 19.7	0.83
	*P*‐value**	<0.001	0.001	0.1***
Procalcitonin	At the start of treatment	4.4 ± 0.81	5.75 ± 0.91	0.27
	At the end of treatment	1.98 ± 0.76	1.15 ± 0.25	0.3
	*P*‐value**	0.003	<0.001	0.398***
Creatinine	At the start of treatment	1.38 ± 0.83	1.68 ± 1.29	0.56
	72 h later	1.68 ± 1.29	1.62 ± 1.39	0.89
	7 days later	1.82 ± 1.19	1.58 ± 1.2	0.46
	At the end of treatment	1.58 ± 1	1.37 ± 0.97	0.45
	*P*‐value**	0.26	0.51	0.44***
Blood urea nitrogen (BUN)	At the start of treatment	27.4 ± 16.5	27.2 ± 22.6	0.97
	72 h later	30.8 ± 18.8	29.1 ± 27.4	0.8
	7 days later	31.1 ± 17.3	28.5 ± 15.8	0.67
	At the end of treatment	24.3 ± 14	24.3 ± 15.8	0.67
	*P*‐value**	0.15	0.68	0.97***

* Significant level of difference among the groups in each time period based on independent sample *t*‐test.

** Significant level of difference within each group based on paired sample *t*‐test.

*** Significant level of difference between the two groups based on repeated measures ANOVA.

Endotracheal aspiration (ETA) culture results 7 days after the treatment initiation were negative in nine patients (32.1%) of the inhaled colistin group and in 25 patients (92.6%) of the inhaled tobramycin/fosfomycin group, and the difference among the groups was significant (*P* < 0.001).

The mean duration of treatment was 13.73 ± 3.22 days in the colistin group and 10.85 ± 2.84 days in the tobramycin/fosfomycin group, and the mean treatment duration in the inhaled fosfomycin/tobramycin group significantly was lower (*P* = 0.001).

Two (6.67%) patients in the control group and three (10%) patients in the case group died. In terms of medicine side effects, 11 subjects (39.3%) in the control group and five subjects (18.5%) in the case group exhibited nephrotoxicity symptoms, although the difference was not statistically significant (*P* = 0.09).

## 4 DISCUSSION

*A. baumannii* is one of the most common causes of VAP in the ICUs. Although colistin is the treatment of choice for VAP due to *A. baumannii*, cases of lack of improvement and resistance to colistin have also been reported. On the other hand, some studies have shown that inhaled drugs such as colistin and tobramycin can be helpful in treating VAP. Even though some studies have indicated that nebulization of particular antibiotics, combined with IV usage of broad‐spectrum antibiotics, could speed up VAP recovery, research in this area is limited.^23^ As a result, the purpose of this research was to assess the effectiveness of inhaled colistin versus the combination of inhaled tobramycin/fosfomycin in treating ventilator‐dependent pneumonia caused by XDR *A. bumannii*.

This was the first open‐label randomized controlled trial focusing on the efficacy of inhaled colistin and fosfomycin/tobramycin in treating XDR *A. bumannii‐*caused VAP. The results of the present research showed that both groups did not differ significantly in age and gender distribution, past hospitalization, underlying disease, cause of ICU hospitalization, and laboratory outcomes and had a confounding effect not observed on treatment results. Both colistin and tobramycin/fosfomycin nebulization procedures decrease the treatment duration and raise the improvement rate. On the other hand, tobramycin/fosfomycin nebulization accelerates the recovery of patients. The trend of decreasing CPIS in this group was higher.

Zampieri et al. examined 12 clinical trials on the effectiveness of inhaled antibiotics in treating Stentobacter and *Klebsiella*‐caused VAP. Their results displayed a high effect of 1.23‐fold inhaled antibiotics in VAP treatment. In these 12 trials, *A. baumannii* and *Klebsiella pneumoniae* were the most pathogenic strains, and colistin (nine trials) and aminoglycosides (seven trials, of which three studies evaluated tobramycin) were the most common inhaled antibiotics. In 11 studies, the additive effect of inhaled antibiotics with intravenous antibiotics has been reported.^11^

In clinical trials conducted by Doshi,^9^ Kalin,^24^ Kofteridis,^13^ Korbilla,^25^ and Tumbarello,^26^ a significant effect of inhaled colistin in the treatment of VAP induced by colistin‐sensitive *A. baumannii* has been reported.

In two clinical trial studies conducted by Hallal^12^ and Le Conte,^27^ the inhaled tobramycin–fosfomycin effect in the treatment of *A. bumannii*‐caused VAP was evaluated, and the significant effect of this compound in increasing the incidence of reduced mortality and decreased hospital stay has been shown in the ICU. The tobramycin–fosfomycin effect in treating VAP by other bacteria such as *Klebsiella* has also been confirmed.^14^, ^28^

Wood et al. in 2017 assessed clinical investigations on the impact of inhaled antibiotics on nosocomial pneumonia treatment between 2010 and 2017. Colistin and aminoglycosides, including tobramycin, were the most common antibiotics, and *Pseudomonas* and *Acinetobacter* strains were the most common pathogens. The clinical efficacy of many inhaled antibiotics has been recorded; however, almost half of the studies indicated improved clinical outcomes, and their use was relatively without side effects.^29^

In 2012, Arnold et al. studied the effect of inhaled supplementary colistin and tobramycin in treating 93 subjects with *Pseudomonas aeroginosa* and *A. bumannii‐*caused VAP. This study revealed that despite greater severity of illness, these patients had favorable survival.^30^

Hallal et al. showed that the outcome of patients with *P. aeroginosa* or *A. bumannii* VAP in the inhaled tobramycin group was more satisfactory compared with the intravenous group.^12^

These studies, consistent with our research, show the favorable effect of these two inhaled drugs for adjuvant therapy in treating XDR *A. baumannii*‐caused VAP. As mentioned earlier, fosfomycin is a drug that increases the absorption of tobramycin and other aminoglycosides, which leads to inhibition of protein synthesis and ultimately the killing of bacteria. Therefore, it seems that combination of colistin and tobramycin with fosfomycin can increase the speed of recovery as well as reduce the length of treatment, treatment costs, and the length of hospitalization.

At the same time, our study faced limitations such as small sample size, and therefore, in order to obtain a general and credible conclusion, further studies in this field are necessary. With considering the different interval doses of inhaled agents (the control group was given inhaled colistin 1 million IU every 8 h, whereas the case group was given inhaled tobramycin/fosfomycin 300 mg every 12 h/80 mg every 12 h), the primary outcome (treatment duration) was determined by the investigator who could not blinded to treatment allocation. Therefore, despite the ideality of blinding the study, it was not possible, and the study was conducted in an open‐label format. Also, minimal inhibitory concentrations were not established and is another limitation of the study.

## 5 CONCLUSION

The current study's results suggest that the use of inhaled tobramycin/fosfomycin in cases with XDR *A. bumannii* VAP was associated with a shorter treatment duration in this open‐label study. Further studies are needed.

## AUTHOR CONTRIBUTIONS

Atousa Hakamifard designed the study. Abbas Ali Torfeh Esfahani, Farzin Khorvash, Behrooz Ataei and Saeed Abbasi performed the study. Abbas Ali Torfeh Esfahani collected the data and wrote the first draft of the manuscript. Alireza Homayouni prepared the inhaled fosfomycin. Atousa Hakamifard revised the draft of manuscript. All of the authors have read and approved the final manuscript.

## CONFLICT OF INTEREST STATEMENT

The authors declare no conflict of interest.

## ETHICS STATEMENT

The study was approved by the ethics committee of Isfahan University of Medical Sciences (ethics code: IR.MUI.MED.REC.1398.405).

## Data Availability

The data that support the findings of this study are available from the corresponding author upon reasonable request.
